# Supplementary material for: Optimal Synthesis of Environment-Friendly Iron Red Pigment from Natural Nanostructured Clay Minerals
Source: Nanomaterials (Basel). 2018 Nov 8;8(11):925. doi: 10.3390/nano8110925 (PMC6266117; doi:10.3390/nano8110925)
Supplement: Supplementary file 1 [file nanomaterials-08-00925-s001.pdf]

Optimal Synthesis of Environment-friendly Iron Red Pigment from Natural Nanostructured Clay Minerals

Yushen Lu [1,2], **Wenkai Dong** [1,2], **Wenbo Wang** [1,3,*], **Junjie Ding** [1], **Qin Wang** [1,3], **Aiping Hui** [1,3] and **Aiqin Wang** [1,3,*]

[1] Key Laboratory of Clay Mineral Applied Research of Gansu Province, Center of Eco-material and Green Chemistry, Lanzhou Institute of Chemical Physics, Chinese Academy of Sciences, Lanzhou, 730000, P.R. China; luyushen206@163.com (Y.L.); dongwenkai161@mails.ucas.ac.cn (W.D.); boywenbo@126.com (W.W.); 17693225101@163.com (J.D,); wangqin@licp.cas.cn (Q.W.); aphui1215@163.com(A.H.); aqwang@licp.cas.cn(A.W.)

[2] University of the Chinese Academy of Sciences, Beijing, 100049, P.R. China

[3] Center of Xuyi Palygorskite Applied Technology, Lanzhou Institute of Chemical Physics, Chinese Academy of Sciences, Xuyi 211700, P.R. China

* Correspondence: boywenbo@126.com (W.W.); aqwang@licp.cas.cn (A.W.); Tel.: +86 931 4968118.

I. Supplementary Tables

Table S1. The main crystal phase information of the selected natural clay minerals.

Clay minerals	Phase Information	Formula	2*θ* (°)	*d* (nm)	(h k l)
Illite/smectite (IS)	Illite/smectite		6.08	1.4525	
			8.79	1.0052	001(I)
			19.71	0.4501	002(I)
			34.90	0.2569	003(I)
Rectorite (REC)	Rectorite JCPDS: 29-1495	$K_{1.2}Al_4Si_8O_{20}(OH)_4 \cdot 4H_2O$	3.57	2.4743	001
			7.15	1.2353	002
			17.60	0.5047	005
			19.99	0.4463	100
			25.17	0.3535	007
			28.83	0.3094	008
Kaolinite (KAO)	Kaolinite 1A JCPDS:78-1996 $Al_2(Si_2O_5) \cdot (OH)_4$	$Al_2(Si_2O_5) \cdot (OH)_4$	12.40	0.7131	001
			24.91	0.3571	002
			35.06	0.2556	-201
			35.98	0.2494	-131
	Kaolinite-1Md	$Al_2Si_2O_5(OH)_4$	19.96	0.4443	110
	Muscovite-2M2 JCPDS:70-1869:	$K_{0.77}Al_{1.93}(Al_{0.5}Si_{3.5})O_{10}(OH)_2$	17.78	0.4984	004
			27.96	0.3188	114
Montmorillonite (MMT)	Montmorillonite-15A JCPDS:13-0135	$Ca_{0.2}(Al,Mg)_2Si_4O_{10}(OH)_2 \cdot 4H_2O$	5.78	1.5236	001
			39.56	0.2276	200
	Montmorillonite-15A JCPDS:29-1498:	$Na_{0.3}(Al,Mg)_2Si_4O_{10}(OH)_2 \cdot 4H_2O$	17.47	0.5072	003
			19.89	0.4460	100
			35.11	0.2553	105
			61.88	0.1498	300
Vermiculite (VMT)	Vermiculite		9.26	0.9538	002
			27.83	0.3201	-117
	Biotite		10.56	0.8364	002
Sepiolite (SEP)	Clinochlore-1MIIb	$(Mg_5Al)(Si,Al)_4O_{10}(OH)_8$	6.19	1.4623	001
	Sepiolite	$Mg_8(OH)_4Si_{12}O_{30}(H2O)_{12}$	7.24	1.2198	110
	Talc JCPDS:73-0147:	$Mg_3(Si_2O_5)_2(OH)_2$	9.49	0.9311	001
			18.66	0.4749	002
			28.65	0.3113	003
	Dolomite JCPDS:36-0426:	$CaMg(CO_3)_2$	30.99	0.2883	104
			33.58	0.2660	006
	Calcite, syn JCPDS:86-0174:	$Ca(CO_3)$	23.11	0.3844	112
			29.45	0.3029	104
Halloysite (HYS)	Halloysite-10A JCPDS:29-1489:	$Al_2Si_2O_5(OH)_4 \cdot 2H_2O$	8.91	0.9911	001
			35.03	0.2550	110
	Halloysite-7A JCPDS:29-1487:	$Al_2Si_2O_5(OH)_4$	12.02	0.7304	001
			19.98	0.4438	100
			24.66	0.3606	002
			62.41	0.1486	300
Illite (ILL)	Illite-2M1 [NR] JCPDS:26-0911:	$(K,H_3O)Al_2Si_3AlO_{10}(OH)_2$	8.83	1.0070	002
			19.84	0.4470	110
			28.08	0.3147	114

Table S2. The main FTIR absorption bands of the selected natural clay minerals.

	**Band position (cm^{-1})**							
Assignment	IS	REC	KAO	MMT	VMT	SEP	HYS	ILL
M-O-H Str.	3699	3639	3698	--	--	3675	3699	--
M′-O-Si-O-H Str.	3620	--	3621	3623	--	--	3624	--
O-H Str. (H_2O)	3424	3422	3425	3423	3431	3435	3437	3434
H-O-H Ben. (H_2O)	1631	1631	1629	1636	1633	1630	1630	1633
Si-O Str.	--	--	1103	--	--	--	1094	--
Si-O-Si Str.	1034	1051	1036	1036	1012	1019	1034	1027
Al-OH Str.	915	910	912	916	--	--	911	--
Si-O_4 (SiO_2)	794	--	791	794	--	--		796
Si-O Str. (SiO_2)	694	696	691	--	--	--	686	--
Si-O-Al Str.	531	547	538	520	--	--	535	522
Si-O-Si Ben.	470	437	469	467	442	445	468	468

Str.- Stretching vibration.

Ben.- Bending vibration.

M, M′ -Al, Mg or Fe

Table S3. Structural information of the selected clay minerals and the as-prepared hybrid pigments, and the a*-values of hybrid pigments.

	Clay minerals			Hybrid pigments		
	Morphology	Type	ξ	Diffraction peaks	α-Fe_2O_3 morphology	a^*-value
IS	Sheet	Mixed-layer	Variable	Disappeared	Nanoparticles	10.4
REC	Sheet	Mixed-layer	Variable	Weakened	Nanoparticles/ Nanotubes	10.9
KAO	Sheet	1:1	~0	Weakened	Nanoparticles	12.1
MMT	Sheet	2:1	~0.2-0.6	Weakened	Nanoparticles / Nanotubes	15.4
VMT	Sheet	2:1	~0.6-0.9	Disappeared	Nanoparticles/ Nanotubes/ Litchi-like microspheres	20.2
SEP	Fibre	2:1	--	Disappeared	Litchi-like microspheres	22.0
HYS	Hollow tube	1:1	--	Disappeared	Nanoparticles	29.6
ILL	Sheet	2:1	~0.6–1.0	Weakened	Nanoparticles	35.2

ξ : net layer charge per formula unit

II. Supplementary Figure

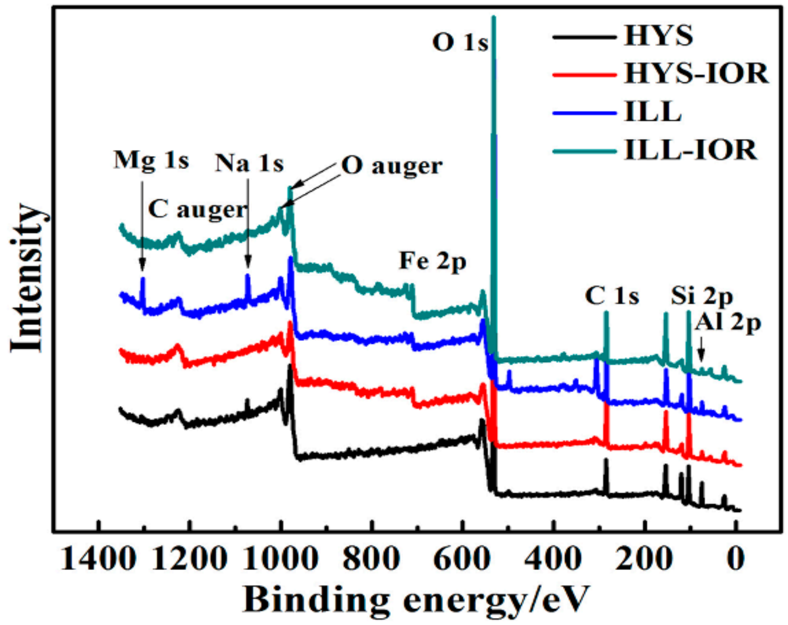

Figure S1. XPS full scanning spectrum of HYS, ILL, HYS-IOR and ILL-IOR.

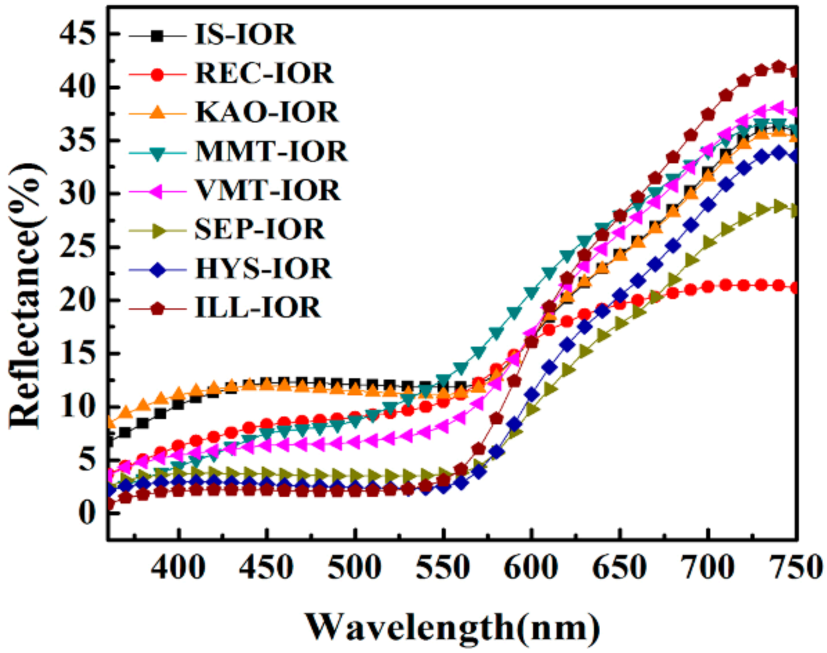

Figure S2. UV–vis diffuse reflectance spectra of the iron-red hybrid pigments.

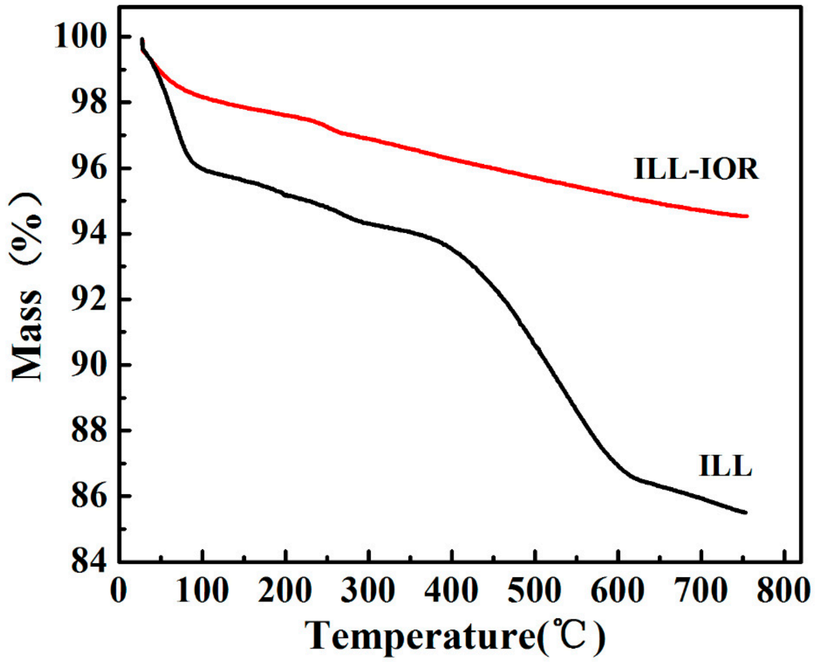

Figure S3. TGA curves of ILL and ILL-IOR.

As can be seen from TGA curves of ILL and ILL-IOR (Figure S3), the weight loss process of ILL is divided into two parts: (i) below 400 °C, it loses the adsorption water and the interlayer water, weight loss about 6%. (ii) above 400 °C, the illite dehydrates the hydroxyl group, weight loss about 9%. What's more, ILL-IOR weight loses about 6% during the whole process, which is close to the first stage of ILL, and there is no obvious weight loss step. It indicates that the mass loss in ILL-IOR may be due to the loss of adsorbed water and interlayer water by illite. ILL-IOR has less quality loss, which proving that ILL-IOR has good thermal stability from the side.
